# Metastatic Melanoma Presenting as Multiple Cardiac Masses: The Critical Role of Endomyocardial Biopsy

**DOI:** 10.1016/j.jaccas.2025.105138

**Published:** 2025-09-24

**Authors:** Teresa Bernardes, Brigh Turner, Alexander Kong, Man Zhang, Eamon Byrnes, Wang L. Cheung, Yahaira Ortiz, S.J. Carlan

**Affiliations:** a Department of Internal Medicine, Orlando Regional Healthcare System, Orlando, Florida, USA; b Department of Cardiology, Orlando Regional Healthcare System, Orlando, Florida, USA; c Department of Pathology, Orlando Regional Healthcare System, Orlando, Florida, USA; d Academic Affairs and Research, Orlando Regional Healthcare System, Orlando, Florida, USA

**Keywords:** cancer, heart failure, melanoma

## Abstract

**Background:**

Tachycardia-induced cardiomyopathy (TICM) is typically reversible with rhythm control, but individual susceptibility remains poorly understood and may reflect genetic predisposition.

**Case Summary:**

A 66-year-old woman with paroxysmal atrial fibrillation (AF) presented with new-onset heart failure. Genetic testing identified a likely pathogenic heterozygous *ABCC9* gene variant (c.3892+2T>C), not previously associated with dilated cardiomyopathy or AF. *ABCC9* loss-of-function mutations have been linked with cardiac channelopathies and cardiomyopathies. Ventricular function improved with rhythm control and medical therapy.

**Discussion:**

This case illustrates the role of *ABCC9* mutations in arrhythmia-induced cardiomyopathy beyond pure TICM. This variant has not been previously reported in individuals with this condition. The co-occurrence of prolonged QT interval, familial AF, and dilated cardiomyopathy underscores the value of genetics in cardiac disease.

**Take-Home Messages:**

Genetic testing may reveal causes in atypical cardiomyopathies and arrhythmias. This novel *ABCC9* variant suggests a genetic contribution to AF-induced cardiomyopathy beyond the expected course of TICM.

## Graphical Abstract

**Figure:**
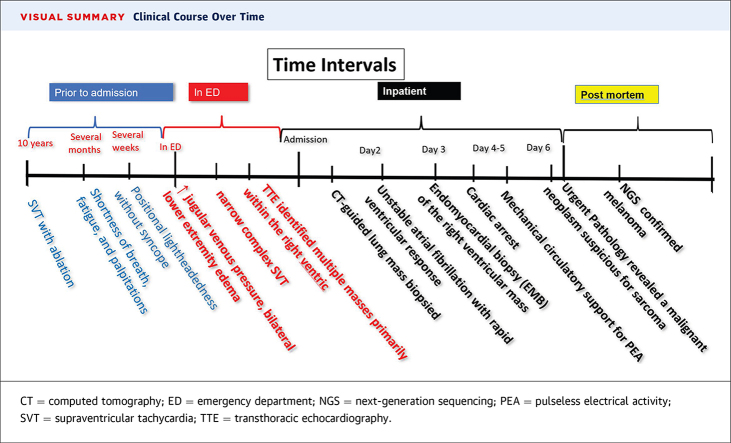

## History of Presentation

A 60-year-old woman with a history of supraventricular tachycardia (SVT) treated with ablation 10 years ago presented to the emergency department of an outside facility with worsening shortness of breath, fatigue, and palpitations over the past few months. She also reported positional lightheadedness without syncope in recent weeks. Her symptoms resembled those experienced before SVT ablation, although she had remained asymptomatic until this visit. Her condition had significantly impacted her ability to work as a housekeeper.

**Take-Home Messages**

• The use of multimodal imaging is critical in evaluating cardiac masses, but biopsy is the gold standard.
• Next-generation sequencing analysis of biopsy tissue specimens may be necessary for a definitive confirmation of cardiac melanoma.
• Management strategies of cardiac melanoma depend on symptom severity and underlying pathology as well as metastatic burden.

She had no history of smoking, but experienced chronic exposure to secondhand smoke from her spouse. She denied having any significant family history of cardiovascular disease.

**Figure 1:**
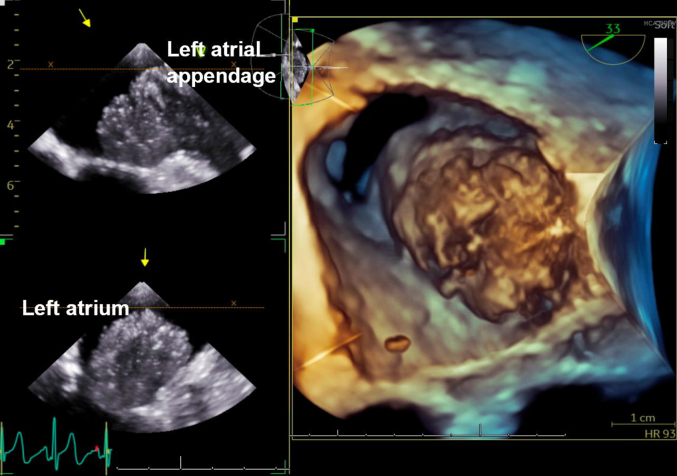
Transesophageal Echocardiography 3-Dimensional Image Showing a Large Mass Originating From the Left Atrial Appendage Without Crossing the Mitral Valve

On physical examination, the patient was tachycardic with a heart rate reaching 160 beats/min, increased jugular venous pressure, and bilateral lower extremity edema. The electrocardiogram at admission demonstrated a narrow complex SVT. Laboratory findings revealed elevated brain natriuretic peptide level of 1,186 pg/L (normal: less than 100 pg/mL). Computed tomography (CT) scan of the chest showed multiple pulmonary and intracardiac masses. Transthoracic echocardiography revealed multiple masses primarily located in the right ventricle as well as a large mass in the left atrial appendage extending toward but not crossing the mitral valve (Figure 1).

## Differential Diagnosis

At this point, the differential diagnosis included primary cardiac tumors and metastatic cardiac tumors. Inflammatory masses such as infectious endocarditis and sarcoidosis were also considered, along with thrombus complications and valvular complications. She was started on therapeutic anticoagulation, broad-spectrum antibiotics, and rate control using metoprolol and flecainide for SVT.

## Investigations

**Figure 2:**
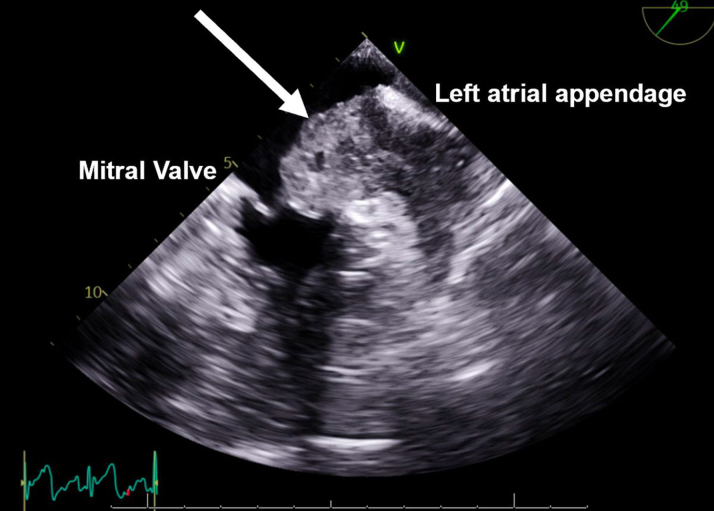
Transesophageal echocardiography Showing a Left Atrial Appendage Mass Without Crossing the Mitral Valve The arrow points to the left atrial appendage mass.

**Figure 3:**
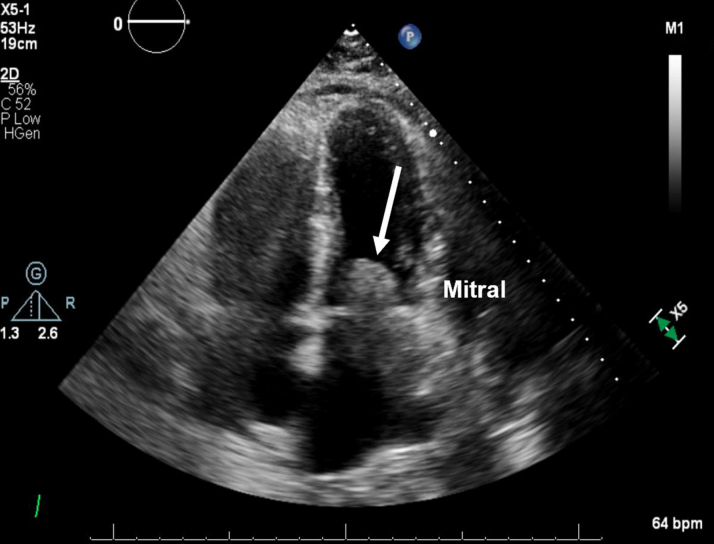
Transthoracic Echocardiography at Day 20 Showing a Rapidly Growing Obstructive Mass Crossing the Mitral Valve From the Left Atrium Into the Left Ventricle Cavity The arrow points to the obstructive mass.

**Figure 4:**
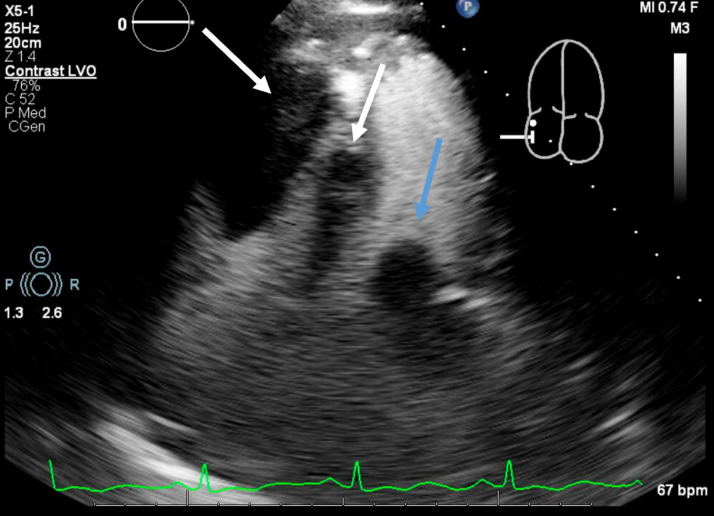
Transthoracic Echocardiography at Day 20 With Contrast Echo Demonstrating Right Ventricular Masses and a Left Atrial Mass The white arrows point to the right ventricular masses, and the blue arrow points to the left atrial mass.

**Figure 5:**
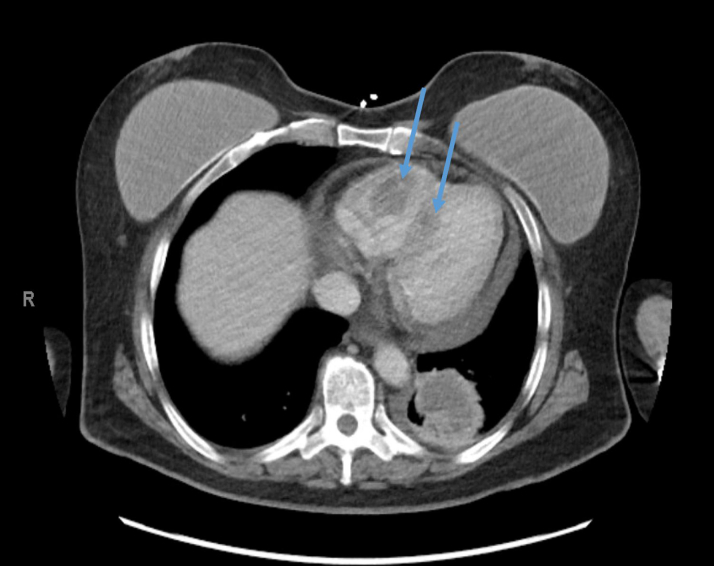
Computed Tomography of the Chest at Day 20 Showing Multiple Hyperintense Intracardiac Masses The blue arrows point to the intracardiac masses.

A CT-guided biopsy of the lung mass was inconclusive. She was transferred to our cardiac critical care unit for a multidisciplinary evaluation involving cardiothoracic surgery, electrophysiology, advanced heart failure, pulmonology, critical care, and oncology teams. Repeat imaging (Figure 2, Figure 3, Figure 4, Figure 5) showed progressive intracardiac masses that were obstructing mitral valve inflow, with the mass extending from the left atrial appendage through the mitral valve.

Because her pronounced presyncopal symptoms with supination led to hemodynamic compromise, cardiac magnetic resonance imaging (MRI) was considered unsafe. Given the high suspicion of malignancy and the need for surgical planning, an endomyocardial biopsy (EMB) of the right ventricular mass was performed with the patient seated at a 90° angle. The procedure was carried out with cardiothoracic surgery backup and extracorporeal membrane oxygenation on standby.

## Management

While waiting for biopsy results, the patient developed unstable atrial fibrillation with rapid ventricular response and obstructive shock, raising concerns for worsening mitral valve inflow obstruction. She was started on phenylephrine and amiodarone infusions but later developed bradycardia and experienced a pulseless electrical activity arrest. Return of spontaneous circulation was achieved after 4 minutes of cardiopulmonary resuscitation. She was intubated and put on mechanical ventilation. Shortly after, she experienced a second pulseless electrical activity arrest lasting 6 minutes, followed by subsequent return of spontaneous circulation.

## Outcome and Follow-Up

**Figure 6:**
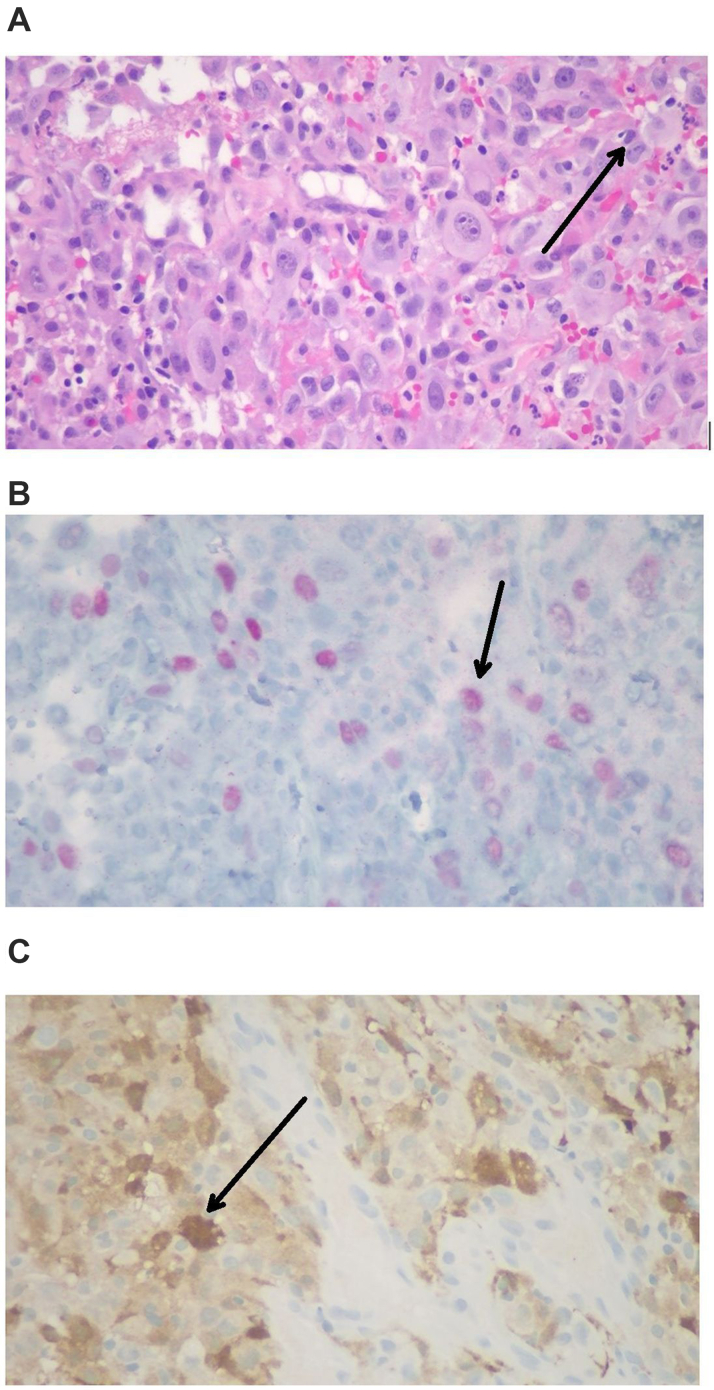
Malignant Neoplasm Suspicious for Sarcoma (A) Hematoxylin and eosin–stained section shows malignant cells infiltrating the cardiac tissue with pink cytoplasm, mildly pleomorphic nuclei, and discrete nucleoli (black arrow). The tumor cells are arranged in sheets with areas of necrosis and scattered mitotic activity. (B and C) Immunochemistry staining shows the tumor cells are weak focal SOX-10 positive (B) and patchy S-100 positive (C).

An urgent pathology review revealed a malignant neoplasm suspicious for sarcoma (Figure 6). Given the prognosis, mechanical circulatory support was not pursued. After discussions with her spouse, her code status was changed to do not resuscitate, and she was transitioned to comfort care. She died with family at her bedside.

Postmortem pathology confirmed BRAF V600E-mutated melanoma. The pathology indicated that the infiltrating tumor cells in cardiac tissue were patchy positive for S-100 and weakly focal positive for SOX-10. The alpha-smooth muscle actin showed weak scattered positivity. The tumor cells tested negative for MART-1, HMB-45, desmin, myogenin, MyoD1, pan cytokeratin AE1/3, CAM5.2, CD34, CD45, and CD20. CD68, CD31, and CD43 marked background histiocytes, whereas CD3 highlighted scattered background T cells. IN1 showed intact nuclear staining. The immunostaining profile of the tumor was nonspecific, and the overall findings suggested a malignant undifferentiated neoplasm, possibly representing an undifferentiated pleomorphic sarcoma. Next-generation sequencing analysis of the specimen by Genexus (Thermo Fisher Scientific) revealed a BRAF V600E mutation, confirming a diagnosis of cardiac melanoma.

The primary malignant melanoma tumor was never discovered.

## Discussion

Melanoma is an aggressive malignancy that originates from melanocytes, the pigment-producing cells found in the epidermis. It is the fifth most common cancer in the United States, with an increasing incidence over recent decades.^1^ The primary risk factor for melanoma is exposure to UV radiation, whether from natural sunlight or artificial sources such as tanning beds. Genetic predispositions, fair skin, and immune suppression also play a role in its pathogenesis.^2^^,^^3^

Although melanoma accounts for only 1% of all skin cancers, it is responsible for more than 80% of skin cancer–related deaths owing to its high metastatic potential.^1^ Melanoma can metastasize to nearly any organ, with cardiac involvement being a rare but significant manifestation. In autopsy studies, cardiac metastases were identified in 64% of patients with metastatic melanoma; however, they are diagnosed in <2% of living patients.^4^, ^5^, ^6^ The underdiagnosis of cardiac melanoma metastases in vivo is attributed to its often silent or nonspecific presentation.

Cardiac masses can lead to significant hemodynamic compromise and arrhythmias, whether they are benign or malignant. Their clinical manifestations largely depend on their location within the heart. The differential diagnosis for intracavitary cardiac masses includes benign tumors (eg, myxomas, lipomas, rhabdomyomas), malignant primary tumors (eg, sarcomas), metastatic lesions, and intracardiac thrombi.^7^ Given the rarity of primary cardiac malignancies, metastatic disease should be heavily considered in cases involving multiple cardiac masses.^7^

Historically, EMB was the gold standard for diagnosing cardiac tumors; however, it has largely been supplanted by a structured multimodal imaging approach when feasible. This approach includes echocardiography, cardiac MRI, and positron emission tomography scans, all of which assist in differentiating malignancies from benign lesions or thrombi.^7^ Nonetheless, EMB remains essential in cases where imaging findings are inconclusive or when histopathologic confirmation is necessary for treatment planning.

This case highlights the diagnostic and therapeutic challenges of intracardiac metastatic melanoma. Cardiac metastases are often asymptomatic, but can lead to significant hemodynamic and electrical instability when they develop in critical locations.^5^ The presence of multiple intracardiac masses should raise suspicion for metastatic disease, particularly in patients with a history of malignancy. Owing to its rarity, cardiac metastasis from melanoma is often diagnosed late, which contributes to poor outcomes.^6^

When melanoma metastasizes to the heart, there is usually only a single mass; however, 13% of patients with cardiac metastases have multiple cardiac tumors.^4^ This patient presented a rare case of multiple cardiac masses at the time of diagnosis.

The use of multimodal imaging is critical in evaluating cardiac masses. Echocardiography is typically the first-line modality, but cardiac MRI and combined positron emission tomography/CT are often necessary for further tissue characterization.^4^ Establishing the nature of a cardiac mass is essential for determining treatment options. Although multimodal imaging frequently allows for diagnosis without biopsy, EMB remains crucial in selected cases. In patients intolerant to supination or with suspected melanoma, EMB is particularly helpful, as melanoma may resemble benign lesions on MRI, such as lipoma or acute thrombus, owing to the T1-shortening effects of melanin.^8^

Management strategies depend on the severity of symptoms and the underlying pathology. In cases of obstructive shock caused by tumor burden, interventions such as surgical debulking, systemic therapy, or extracorporeal membrane oxygenation may be considered.^9^ However, advanced metastatic disease often prevents aggressive intervention, necessitating discussions about palliative care.^1^

The presence of a BRAF V600E mutation suggests potential responsiveness to targeted therapies, but the rapid clinical decline of the patient in this case prevented treatment initiation.^10^ This case underscores the importance of early recognition of cardiac metastases and highlights the role of a multidisciplinary team in managing complex oncologic and cardiologic emergencies.

## Conclusions

Advancements in cardiac multimodality imaging have made it possible to diagnose and guide the treatment of cardiac masses without biopsy in most cases. However, in hemodynamically unstable patients or when melanoma is suspected, endomyocardial biopsy may be necessary, as melanoma can mimic benign lesions on cardiac MRI owing to the paramagnetic T1-shortening effects of melanin. This case underscores the challenges of diagnosing and managing cardiac masses in the setting of malignancy and highlights the critical role of a multidisciplinary approach in optimizing care for critically ill patients.

**Visual Summary:**
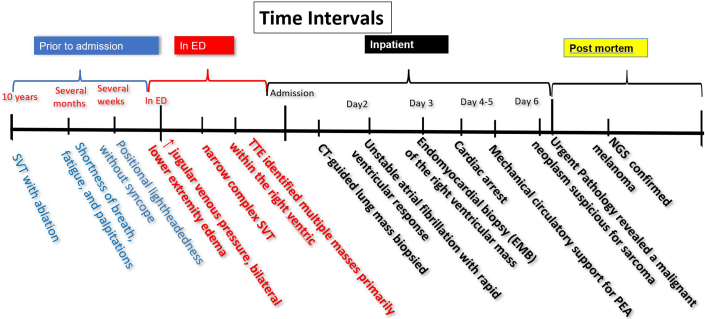
Clinical Course Over Time CT = computed tomography; ED = emergency department; NGS = next-generation sequencing; PEA = pulseless electrical activity; SVT = supraventricular tachycardia; TTE = transthoracic echocardiography.

## Funding Support and Author Disclosures

The authors have reported that they have no relationships relevant to the contents of this paper to disclose.
